# Pathways to opioid use and implications for prevention: voices of young adults in recovery

**DOI:** 10.1186/s13011-023-00584-5

**Published:** 2024-01-18

**Authors:** Parissa J. Ballard, Taylor J. Arnold, Elena M. Vidrascu, Guadalupe C. Hernandez, Emily Ozer, Mark Wolfson, Rebekah Lassiter, Himani Nayyar, Stephanie S. Daniel

**Affiliations:** 1 Wake Forest University School of Medicine, 1920 West 1st St, Winston-Salem, NC 27104 USA; 2 Psychology & Neuroscience, University of North Carolina at Chapel Hill, Chapel Hill, NC USA; 3 School of Public Health, University of North Carolina, Chapel Hill, NC USA; 4 Community Health Sciences, School of Public Health, University of California Berkeley, Berkeley, CA 94720 USA; 5 Department of Social Medicine, Population, and Public Health, University of California, Riverside, CA 92521 USA; 6 Wake Forest University, Winston-Salem, NC USA

**Keywords:** Opioid use disorder, Adolescents and young adults, Substance use prevention

## Abstract

**Background:**

Opioid use remains a major public health issue, especially among young adults. Despite investment in harm reduction and *supply-side* strategies such as reducing overprescribing and safe medication disposal, little is known about *demand-side* issues, such as reasons for use and pathways to opioid use. Adolescents and young adults who struggle with opioid use disorder (OUD) are multifaceted individuals with varied individual histories, experiences, challenges, skills, relationships, and lives.

**Methods:**

To inform the development of prevention strategies that hold promise for addressing opioid use, this study employs brief structured surveys and semi-structured in-depth interviews with 30 young adults (ages 18–29; 19 female, 23 White, 16 from Suburban areas) in recovery from OUD. For survey data, we used descriptive statistics to summarize the means and variance of retrospectively reported risk and protective factors associated with opioid use. For in-depth interview data, we used a combination of thematic analysis and codebook approaches to generate common themes and experiences shared by participants.

**Results:**

Surveys revealed that the most endorsed risk factors pertained to emotions (emotional neglect and emotional abuse) followed by sexual abuse, physical abuse, and physical neglect. Themes generated from qualitative analyses reveal challenging experiences during adolescence, such as *unaddressed mental health, social, and emotional needs*, which were often reported as reasons for opioid initiation and use. Through surveys and interviews, we also identified positive assets, such as *skills* and *social relationship*s that were present for many participants during adolescence.

**Conclusion:**

Implications include the need for universal prevention strategies that include emotion-focused interventions and supports alongside current harm reduction and environmental strategies to regulate prescriptions; the potential utility of more emotion-focused items being included on screening tools; and more voices of young people in recovery.

**Supplementary Information:**

The online version contains supplementary material available at 10.1186/s13011-023-00584-5.

## Background

Adolescents and young adults (AYAs) who struggle with opioid use disorder (OUD) are multifaceted individuals with varied histories, experiences, challenges, skills, relationships, and lives. Although their stories are individual, it is useful to search for similarities and differences from their accounts of their pathways to the non-medical use of prescription drugs and/or use of illicit opioids (hereafter referred to as "opioid use"). These insights can inform prevention intervention and policy that hold promise for diverse youth, given the devastating effects OUD can have for young people and their families.

 The opioid epidemic is a global threat to the healthy development of AYAs. We use the term AYAs to refer to young people between the ages of 12–29 and use the terms “youth” and “young people” interchangeably; we specify age ranges when citing statistics and referring to particular sets of young people. In the United States (US), opioid use remains a major public health issue, especially among young adults. In 2021, 9.2 million people above age 12 in the US used opioids in the past year; the largest percentage were young adults aged 18–24 [1]. There has been broad public, scholarly, and financial investment in understanding opioid use. Research about opioid use disorders among AYAs has focused almost exclusively on understanding the risk pathways that stem from prescription opioid use. Public policy and financial investments have also focused on “supply side” prevention strategies to regulate prescriptions [2] and educate individuals about safe prescription storage and disposal [3] harm reduction strategies, such as naloxone awareness and distribution. These supply-side approaches, such as limiting the quantity of medications prescribed for pain, curbing access through medication disposal programs, and reducing harm, are critical (see [4]). However, applying broader public health and prevention perspectives, particularly focused on the social determinants of opioid use, is necessary to better understand and stem the opioid crisis [2, 5]. In a call to action for physicians, Dasgupta, Beletsky and Ciccarone underscore the need to attend to the root causes driving the demand for opioids, such as physical and psychological trauma, economic disadvantage, isolation, and hopelessness [5]. They suggest using the frame of *suffering* to emphasize root causes instead of focusing on *pain*, which tends to emphasize physical symptoms, as an approach to guide both patient- and community-level interventions [5]. Fraser and Plescia [2] further call for examination of the demand for opioids and bolstering primary prevention, arguing that addressing OUD and addiction requires an “honest and direct examination of the reasons individuals use drugs in the first place”. Especially given that the risk environment for opioids is evolving and becoming more dangerous due to the opioid supply being increasingly adulterated with other compounds such as fentanyl [6], effective prevention approaches are urgently needed. To contribute to understanding the various pathways to opioid use among AYAs with the intent to inform policy and community-level prevention interventions, the present study examines retrospective accounts of young adults (YAs) who identify as beingin recovery from OUD or no longer using opioids.

### Substance use, substance use disorders, and OUD

Substance use is a major health vulnerability during adolescence and the transition into adulthood [7–11]. Opioid use is especially common among AYAs [1]. Opioids include illegal substances such as heroin and street fentanyl, and legal prescription pain relievers such as oxycodone, hydrocodone, codeine, morphine, fentanyl, and many others [12]. Early initiation of opioid use increases risk trajectories [13], which underscores the need to examine opioid use among AYAs.

### Prevention through a risk and protective factor framework

A common guiding framework in prevention science is the risk and protective factor framework which emphasizes the multiple conditions and factors that increase and decrease the likelihood of substance use and related problematic outcomes [14–17]. From a social ecological perspective, risk and protective factors occur across multiple contexts or levels of the social ecology of young people such as the community (e.g., neighborhood), institutions (e.g., school), interpersonal (e.g., family, peer), and individual (e.g., attitudes, genetic factors) levels. Risk factors for general substance use in adolescence and young adulthood are well-characterized across multiple levels [18]. A recent systematic review documented individual-level risk factors, including maltreatment, psychiatric disorders, perceived drug accessibility, high impulsivity, and rebelliousness and individual protective factors such as trait optimism and mindfulness. Family-level risk factors include low parental education, negligence, poor supervision, and presence of substance-using family members while parental knowledge about substance use was a protective factor [16]. In addition, having peers who use drugs was identified as a risk factor while religious beliefs, school connectedness, and presence of supportive adults were protective factors [16].

Many of these risk and protective factors for substances use may also apply to opioid use among adolescents and young adults (AYAs). Although few studies have examined risk factors associated with opioid use specifically, there is some evidence of risk factors for opioid use such as: emotion regulation difficulty, previous substance use, delinquent behavior, low mindfulness, ease of access, having a close friend who used other substances, parents with alcohol use disorders, living in a rural community, disconnectedness from school and job opportunities, and lack of homework completion [15, 16]. The current opioid epidemic affects young people from diverse demographic backgrounds across social class, race/ethnicity, and biological sex [19]. A review of prescription drug use among US racial/ethnic minorities found that physical pain, mental illness, and stress significantly predicted use [19]. Some studies suggest differences in substance use based on educational attainment, with non-medical use of prescription opioids more common among those disconnected from school, and other substance use (e.g., non-medical use of prescription stimulants) more prevalent among those connected to higher education (e.g., college students and graduates) [20]. Despite some evidence about risk factors, much remains unknown about how such factors confer risk, how they function in the lives of young people, and how they might be mitigated.

### Pathways to opioid use

The risk and protective factos framework is widely used to predict substance use outcomes, particularly in quantitative research. However, this framework often fails to discuss the *complex interplay* between risk and protective factors, which shape people’s path toward substance use. Qualitative methods enable us to capture this complexity more fully, especially through narrative approaches, as they provide valuable insights about individuals’ lived experiences through their own perspectives and stories [21]. Past qualitative research examining opioid use has focused on experiences of current users to a greater extent than on those in recovery [23]. Hearing from young people *in recovery* is an important source of firsthand information about paths to OUD that responds to calls to amplify the voices of people most affected by the issue being studied [24]. Qualitative approaches are especially powerful to understand the perspectives of AYAs in recovery from OUDs and their accounts of the ecology of their lives and the factors that shape their pathways to OUD [22, 23]. With respect to opioid use specifically, little is known about risk factors that contribute to OUDs such as the use of opioids recreationally and/or to cope with problems, or underlying psychosocial risk factors. One study examining opioid use initiation among adults revealed three main motives: coping with mental health and stressors, physical pain relief, and experimentation [22]. Drawing on and extending the risk and protective factor framework, in the present study we consider individual risk factors instead as “microsocial factors” (see [24]), or potential signals of social problems and signals that young people in distress need support. This language is meant to highlight opioid use as a complex and multi-level problem and not overemphasize the individual’s role and responsibility or, at the extreme, blame individuals for not “better” dealing with adversities and challenges. To extend current knowledge about pathways to opioid use among adolescents and young adults, a qualitative approach to understanding microsocial factors and pathways from the stories from young adults (YAs) in recovery from OUD can help conceptualize the complex factors involved.

### The present study

Through both surveys and interviews, the present study draws on retrospective accounts of young adults in recovery from opioid use disorder (OUD) from a state in the Southeastern US. Our goal was to understand their perspectives on their own pathways to opioid use by reflecting on their substance use in adolescence and beyond. Specifically, this study sought to describe the challenging experiences that YA participants in recovery from OUD described from their adolescence, around the time they started using substances, as well as the assets in their lives.

## Methods

### Study design

The study uses both quantitative and qualitative data; quantitative data describe mean levels of risk and protective experiences and qualitative data explore pathways to OUD using narrative accounts. The study was conducted under the purview of the Institutional Review Board at the Wake Forest University School of Medicine (IRB00054756). Qualitative study procedures are reported according to the Consolidated Criteria for Reporting Qualitative Research (COREQ) checklist [25].

### Sample

The sample consists of 30 YAs. In our screening form, participants were asked “Thinking about your current or past opioid/non-medical prescription opioids use, which best describes you” with the options “currently using, not currently using, in recovery.” All participants self-identified as either “not currently using” opioids (n = 2) or being “in recovery” (n = 28) and all identified a date they considered the start of the period of recovery for them. There were many different recovery pathways followed (e.g., Twelve Steps, Refuge Recovery); we did not inquire whether participants followed abstinence or medication-assisted recovery. As inclusion criteria, we required that participants had a close social support network and/or a mental health provider that they could follow up with for support if needed at any point throughout the study in the event that information brought up in interviews was difficult for participants and necessitated support; no participants were excluded based on this criterion. The largest proportion of participants identified as female, White, and from suburban areas (see Table 1). Inclusion criteria were being between the ages of 18 to 29 and identifying that opioids had presented them with problems in their life. All 30 participants reported that opioid/non-medical prescription opioid use had presented problems for them, with 27 participants reporting additional substances that had been problematic for them (see Table 2 for more information related to substance use and recovery). Interviews were only conducted once and findings were shared with participants (see below).

**Table 1:** Sample demographics

Gender Identity*			
Male			10
Female			19
Non-binary			1
Race/ethnicity			
White			23
Black or African American			3
Hispanic, Latino or Spanish origin			1
American Indian or Alaskan Native			3
AAPI			2
More than 1 race			2
Born in USA			27
Not born in USA			3
Age			
18–21			3
22–25			10
26–29			17
Geographic area			
Urban			11
Suburban			16
Rural			3
Parental education			
High School/GED			7
Some College			7
College Degree			12
Graduate Degree			4

*Note.* Intending to use inclusive language, we asked participants about “gender identity” with the options “male, female, trans male/trans man, trans female/trans woman, genderqueer/Gender non-forming, non-binary, different identity, and prefer not to answer”. Following modern guidance [55], we would now use the terms “man” and “woman” instead of "male" and "female"

**Table 2:** Substances, access, and recovery information

	N			
Substances identified as problematic in the past				
Opioids	30			
Alcohol	15			
Cannabis	16			
Tobacco or E-cigarettes	15			
Cocaine	19			
Stimulants	16			
Tranquilizers	19			
Hallucinogens	11			
Access^a^				
Friends	36.2%			
Romantic partners	19.1%			
Family	17.3%			
Personal prescriptions	16.4%			
Other people’s medicine cabinets	12.2%			
Recovery				
Recovery coach or sponsor	25			
Mental health provider	21			
Months in recovery	Mean	(SD)	Min	Max
	29.43	(24.31)	1	86

^a^percentages of each "source of access" code out of the total number of access codes across all 30 interviews

### Data collection

Participants were recruited using a network-based approach, posting study flyers on recovery-related online groups and platforms, through formal recovery programs, and through a snowball sampling technique by which participants were recruited by their friends. Recruitment efforts continued until the recruitment goal of N = 30 participants was met. A total of forty-two participants were recruited and screened, with 30 remaining eligible after excluding 5 for being outside the required age range (18-29), 3 for not selecting opioids as having caused them problems in their life, and 4 for lost communication.

Participants completed an electronic screening form (via REDCap) to confirm eligibility and if eligible, were contacted to schedule interviews that were part of a larger study (PI Ballard, funded by the National Institute on Drug Abuse). Semi-structured interviews were conducted via WebEx or Zoom videoconferencing between May and October 2021 in a one-on-one setting with both the interviewer and interviewee in the private space of their respective homes or offices. Three people conducted interviews (the PI and two study team members). The two study team members received training from the PI in the conduct of interviews, including interviewing about sensitive topics, as well as the content of the interview. Training included didactic components, practice interviews, listening to the recording of the first interview (conducted by the PI), and receiving feedback on their first recorded interview, as well as ongoing discussion about issues that arose as the interviews were conducted. The interviews ranged from 15 to 90 min depending on the length of participant responses and all participants agreed to be audio recorded. Recordings were professionally transcribed. Transcripts were not returned to participants for review; some participants provided feedback on these findings via feedback sessions (see Positionality Process section below for details) focused on ensuring that we were using sensitive language and that we invited input on the main themes. Self-administered surveys were completed electronically via RedCap immediately following the interviews. Participants were compensated with a $50 gift card for participation in the study, and an additional $25 gift card for participation in the follow-up feedback session.

### Measures

#### Quantitative

Demographics, individual characteristics, and risk and protective factors were assessed via self-report questionnaires. *Demographic characteristics* included age, race/ethnicity, gender identity, and parental education. *Risk factors* included the 28 item Childhood Trauma Questionnaire [26] assessing emotional, physical, and sexual abuse, and both emotional and physical neglect. *Protective factors* were measured through the 17-item Child and Youth Resilience Measure - Person Most Knowledgeable [27, 28] assessing personal and caregiver factors that may bolster resilience, as well as 3 items assessing contribution to community [29] and 4 items assessing connection and contribution to community [30]. We also collected data  related to recovery (e.g., length and available support).

#### Qualitative

Semi-structured interviews followed our interview guide (Appendix A). Part 1 of the interview elicited participants’ description of their current life; Part 2 elicited reflections regarding participants’ substance use and specifically their experiences with opioids; Part 3 asked participants to describe their experiences with recovery, their suggestions for prevention, and the potential roles of young people in prevention efforts.

### Data analysis

#### Quantitative

We conducted descriptive statistical analysis of the surveys to summarize the means and variance of retrospectively reported risk and protective factors associated with opioid ues. We created composite scores of the subscales of each measure, computed scale reliabilities using Cronbach’s alphas, and computed means and variance (Table 3).

#### Qualitative

Interviews were analyzed through a series of steps following both thematic and codebook approaches. First, we approached interview data through a thematic analysis following six phases of: familiarizing ourselves with the data, generating initial codes, searching for themes, reviewing themes, defining and naming themes, and generating a report [31, 32]. Our analysis was both deductive (in that themes were informed by our specific topics of interest) and inductive (in that themes were informed by our data [32]). Three authors (PJB, GCH, EMV) read sets of interviews (10 each) and composed memos to reflect on and get to know the data. Next, these three authors were joined by two additional team members (HN, RL) to determine broad themes that appeared across the interviews and to identify nuances and provide examples and counter examples.

Next, the team refined the list of initial themes generated and created a list of codes in Atlas. Ti computer software. Departing from a thematic analysis approach (now referred to as a “reflexive thematic analysis” [33]), the first and second authors then developed a codebook which included the following about each code: its definition, a guide for “when to use” and “when not to use” the code, and an example of how the code had been used in an interview. Five team members split up interviews in sets of three at a time, with two people independently coding and then merging codes, concluding with a final coded transcript produced through discussion. Team members coded interview sets and met bi-weekly to discuss, refine codes as needed, and update the codebook. After coding all interviews, we generated outputs for each code. The team divided up the reports (with 2 team members reading each one) and wrote a memo summarizing the main ideas, points of convergence and divergence, and interesting examples. The team discussed individual memos and incorporated group feedback into the memos for each report. In this stage of analysis, we discussed the prevalence of the ideas identified in the memos to document how common themes were across the set of interviews. Findings from these memos were organized into the main themes presented in this manuscript (Table 4). In a final stage of analysis, we discussed what was missing from our coding/analysis process; this discussion surfaced the idea that we had not adequately captured the assets discussed by participants. Although our interview protocol did not specifically ask participants to identify the assets or positive characteristics and contexts in their lives, we noted that some were identified by participants during the interview. To honor this fact and document the positive aspects of participants’ lives that they shared, we completed the analysis with a code for “assets” which we used to capture the positive aspects of participants’ earlier lives (around the time they started using substances or earlier). Although many participants described assets in their present day lives during recovery, we only applied this “assets” code to text describing their lives retrospectively around the time they first started using substances; we conducted a separate analysis of assets in recovery (not included in the present paper).

### Positionality process

Three members of the study team (PJB, GCH, EMV) conducted the interviews. The three interviewers identified as women: one Iranian American, one Mexican American, and one European American. Two were affiliated with Wake Forest University School of Medicine; one was (at the time) an assistant professor who holds a PhD in developmental psychology and has extensive experience conducting qualitative research; the other was (at the time) an associate project manager who holds a BA in communication studies with experience and training in qualitative interviewing and analysis. The third is affiliated with the University of North Carolina, Chapel Hill and is a graduate student researcher in the Psychology and Neuroscience department. Our full research team varies along identity dimensions such as age, gender, race/ethnicity, socioeconomic background, the communities and cultures grown up in, and personal/familial experiences with substance use disorders. To address most team member’s lack of personal experience in recovery, several steps were taken to examine individual positionality throughout our research process in order to ensure that our interactions with participants were respectful, and to interrogate potential biases in our data collection and analyses. In advance of conducting interviews and surveys, our team attended a training hosted by Recovery Communities of North Carolina on issues related to language and stigma in working with people in recovery from SUDs. We each reflected on our own positionality, wrote positionality statements, and discussed them as a group according to each members' comfort disclosing personal stories. Throughout the project, we formed a “journal club” that included academic articles, documentaries and podcasts by, and featuring, people in recovery, such as *Anonymous People*, *Generation Found*, and the episode Day 7 on the *Armchair Expert* podcast. As we conducted interviews and analyses, we reflected on the stories we were hearing and our own reactions to them. With the full team who were involved in analyses (PJB, GCH, EMV, HN, RL), we discussed potential biases in our analyses stemming from our own experiences. Finally, we re-contacted participants to garner interest in a feedback session, with the purpose of sharing themes we found across the set of interviews and gathering feedback on our findings. Twenty-two out of the 26 participants contacted expressed interest in the feedback session upon contact via email or text. Contact information was unavailable for 4 participants. We conducted four feedback sessions consisting of 1–4 participants per session. All sessions were conducted virtually using the Zoom interface and lasted anywhere from 40 to 80 min. We used this feedback in two ways: first, we updated our language directly in response to feedback and second, we note specific places below where feedback from these sessions added context to findings from our qualitative analysis.

## Results

Our research goal was to describe the experiences that YAs in recovery report from the time they initiated substance use. We sought to analyze both the challenges and assets in their lives, with attention in qualitative analyses to how they connect substance use initiation to their experiences. We present quantitative data to describe mean levels of risk and protective experiences and qualitative data to explore pathways to OUD using narrative accounts.

### Quantitative

#### Substance use

All 30 participants identified opioids as having presented a problem in their lives; this served as a confirmation of our sampling approach. Twenty-seven of them reported substances in addition to opioids as being problematic for them (Table 2). Participants varied in the length of their current recovery period from 1 to 86 months (avg. 29 months).

#### Risk factors

The most endorsed risk factors pertained to emotions (emotional neglect mean = 3.6; emotional abuse mean = 2.4) followed by sexual abuse, physical abuse, and physical neglect. In terms of access, as coded from interviews, substances were accessed most often through friends, followed by romantic partners and then family, personal prescriptions, and other people’s medicine cabinets (Table 2).

#### Protective factors

The most endorsed protective factors reported were related to caregivers (such as “My parent(s)/caregiver(s) really looked out for me”), followed by personal protective factors (such as “I cooperated with people around me”). The lowest endorsed protective factors were in the domain of connection and contribution to community (Table 3).

**Table 3:** Risk and protective factors

Variable name	# Items	Reliability	N	Mean	SD	Min	Max
Risk factors							
Emotional abuse	5	0.90	30	2.413	1.266	1	5
Physical abuse	5	0.92	30	1.890	1.260	1	5
Sexual abuse	5	0.98	30	2.200	1.604	1	5
Emotional neglect	5	0.92	30	3.580	1.197	1	5
Physical neglect	5	0.83	30	1.790	0.868	1	5
Minimization/denial	3	0.86	30	2.622	1.240	1	5
Protective factors							
Community connection/contribution	7	0.93	30	2.340	1.093	1	5
Resilience - personal	10	0.91	30	2.970	1.059	1	5
Resilience - caregivers	7	0.86	30	3.140	0.996	1	5

### Qualitative

We identified three main themes regarding the challenging experiences that participants described from their adolescence around the time they started using substances. These included: trauma, unaddressed mental health issues, and lack of tools to regulate and cope with emotions (theme 1), negative self-views (theme 2), and social isolation/loneliness/disconnection (theme 3). Where possible, we note the experiences that participants explicitly associated with their substance use. Regarding assets, we identified two main themes: skills/talents (theme 4) and relationships (theme 5). We also noted a theme around characteristics or contexts that were described as both challenges and assets (theme 6; see Table 4). Participant ID numbers are included after quotations to demonstrate the range of participants quoted.

**Table 4:** Themes and illustrative quotes

Theme	Illustrative Quote
**1. Trauma, unaddressed mental health issues and/or difficulty regulating and coping with negative emotions**	*“I’ve always had problems with anxiety, depression. I was very, very sad from a very young age and I think I was just looking for that kind of happiness from outside factors. If it wasn’t drugs, it was guys. I would just use anything I could to feel some type of way.”* [101]
**2. Negative self-view**	*“I always wanted to kind of get out of myself because I didn’t like myself.”* [107]
**3. Social disconnection**	*“I went back to public school technically in the eighth grade, and I was 13 and I didn’t have any friends. No one wanted to be friends with the weird girl from private school. So I didn’t really know anyone or anything, didn’t really have access to anything. I was just really miserable, really alone, and really angry at just the world and everything. All that teenage angst times 20.”* [106]
**4. Skills/talents**	*“… So, as a high schooler, I was running cross country. I was wrestling. Really competitive with pretty much everyone in my class…”* [105]
**5. Relationships**	*“I did have a close relationship. I’ve always been very close with my mom….She is kind of the first person I go to when something goes wrong or anything like that.”* [107]
**6. Simultaneous Challenges/Assets**	*“I was a gifted kid, and so when the time came to actually have to do work and really put time into schoolwork, I was really bad about that, so I skipped a lot of school.”* [104]

#### Challenging experiences

##### Theme 1. Trauma, Unaddressed Mental Health Issues and/or Difficulty Regulating and Coping with Negative Emotions

Many participants reported that at the time they initiated any substance use, they were struggling with earlier experiences of trauma and/or undiagnosed depression, anxiety, and/or bipolar disorder. Participants disclosed traumatic events in the form of physical or sexual abuse and acute events such as a death in the family. For example, one participant described experiencing trauma after a family death and described that they “had no idea how to process it.” This same participant explained, “I think trauma definitely fueled a lot of my addiction…Not everyone who ends up with a substance abuse problem has severe trauma like that in their life, but I’ve found more often than not that’s a really common factor” [106]. Multiple participants described experiences with sexual abuse and connected those experiences to their substance use. For example, one participant shared, “I experienced some sexual abuse when I was a kid…I think that has the potential to have played a factor in it. I know from my experiences [that] trauma has a tendency to go hand-in-hand with addiction and substance abuse” [102]. A different participant had a similar view and felt that holding the secret of a painful experience was connected to their substance use.

“I do think the sexual abuse had a lot to do with that, because I just wouldn’t talk about it. I didn’t tell anyone in my family until I was 26. So I kept that a secret for a really, really long time, and I think that honestly had a lot to do with why I chose to go to opiates. And the fact that my boyfriend died, I just, I kind of didn’t know how to deal with it. I didn’t know how to deal with my own emotions or being me…” [107].

Others shared the same experience and noted that someone suggested to them that their substance use was a form of self-medicating as a result of experiencing trauma; for example, one participant shared, “Right, so at the age of 12, I was molested as well, and there was a huge situation that happened with that.” The participant reflected, “Looking back now, I know I had a lot of things going on as a kid, like mental health-wise. And over time, doctors just told me, ‘Well, you’re self-medicating’ …but in a way, I was trying to escape reality” [118].

Relatedly, within this theme, several participants noted the role of negative emotions and their perceived lack of skills for coping with their emotions as central to their substance use initiation. This theme emerged among both participants who identified later-diagnosed unaddressed mental health disorders and among those who did not report clinically diagnosed mental health issues. For some participants, specific events or experiences were not described explicitly as trauma or abuse, but more broadly, such as instability in their families that often revolved around parental divorce, one or more parents not being present, parental alcohol and substance use, and upheaval related to family re-location or other transitions. Many participants discussed not feeling like they had the tools or coping skills to deal with these events and associated feelings. One participant reflected that “I just, I think, wanted to [use substances] more than feel my feelings and all of that” [114]. Another participant described not knowing how to deal with anger and discomfort, “But I remember being in middle school, and that’s when I started to self-harm and not knowing what to do with my anger and feeling like all this shame and uncomfortable in my own body” [113]. Similarly, another participant explained how they started cutting themselves at a young age, and how opioids were a way of dealing with emotions: “If I wasn’t high, I had all this negative emotion in me and I had no idea how to get it out. But if I got high, then I could cover it all up and everything was okay” [128].

One participant talked about his bipolar disorder (which he stated was undiagnosed for 8 or 9 years) and directly connected his substance use as a form of coping behavior, “It was … a majority of my bipolar comes out as depression…With pills, I could get so numb that I didn’t feel it. With heroin, I could do so much heroin that I didn’t feel” and “Once my bipolar disorder was treated effectively, it was a lot easier for me to stop using drugs” [102]. When participants were asked about resources available, many said they did not feel that they had support or knowledge of any resources. One participant noted having access to a therapist but reported that therapy sessions were exhausting:

“My mom kept enrolling me in therapy. [Laughter] And…I just wouldn’t be honest with them…I feel like it seemed exhausting to try to explain how I felt, and it was easier to just make everything seem okay and just get through it… I didn’t wanna have to share how I felt. I didn’t want to have to open up and try to explain myself, you know, or do any work to make it better. I guess. I just wanted it to go away” [110].

These examples showcase the need for mental health and emotion-focused supports for adolescents, as well as a more general focus on building coping skills.

##### Theme 2. Negative Self-view

One experience that was evident across the interviews was that many participants had a negative view of themselves during their adolescent years. Several people made a direct connection between what they perceived as something missing inside them and using drugs to fill that void. Participants described their negative self-views using phrases like feeling a “lack of peace” with themselves and “not feeling comfortable in their own skin.” The range of valence of negative self-perceptions from mild to intense was striking. Many people described vague and mild discomfort with themselves (not comfortable in own skin, didn’t feel like I fit in, felt lonely). For example, one participant described that “I just always felt uncomfortable like in my own skin and was always just looking for people to make me feel better … I guess like just feeling validated by something or someone” [127]. Another participant was asked about their motivations to use substances and replied “A lot of insecurity, a lot of guilt. Yeah, a lot of just reflection of my past and a lot of negative self-esteem, negative self-worth. Yeah, I think the self-esteem and the self-worth was a huge part of it” [109]. Another participant connected the feeling to opioids explicitly:

“I think that for a long time, I never felt at peace with myself and that’s the feeling that opioids give you. They make you feel like you’re all warm and fuzzy and there’s nothing bad happening in the world at all and you just forget about your problems. And I think that that was the relief that I was looking for” [101].

Others described extreme self-contempt and self-hatred, such as feelings of guilt, shame, anger, and not knowing how to navigate emotions. For example, one participant said:

“I was molested by my cousin. I think I was like six years old…I remember being in middle school, and that’s when I started to self-harm and not knowing what to do with my anger and feeling like all this shame and uncomfortable in my own body. That’s when fighting started in my house and everything, too, was around middle school…. The first time I got high, I was like, ‘This is it. It makes everything go away. I feel good. I feel happy. I’m not stressed out. I’m not worrying. I’m not feeling like everybody’s laughing at me’…I think about like 15-year-old me had so much hatred, so much self-hatred, so much self-loathing, and just contempt for myself. No gentleness in the relationship with myself, no compassion, nothing soft” [113].

As this powerful example illustrates, intense examples of negative self-view often overlapped with a perceived inability to cope with negative feelings, and opioids were described as providing a respite.

##### Theme 3. Social disconnection

Another common theme was a sense of loneliness and social disconnection. Similar to the theme regarding negative self-views, the feelings of social disconnection ranged from mild (e.g., not fitting in, feeling empty) to severe (e.g., not having friends, being bullied). On the mild end, participants described being lonely and wanting more friends. On the more severe end, a participant described being bullied “to the point of deep, deep depression. Like I was on the verge of killing myself…[which was] so traumatic for me that I’ve blocked out… I went through a really dark point where I had like no friends” [115]. These more extreme examples reflected all three themes of social disconnection, negative self-view, and mental health issues/difficulty dealing with emotions as the issues overlapped in their lives with cumulative negative effects. Another example of the cumulative effects of all three of these themes is seen in the following reflection:

“I was really angry, as a kid…But it was kind of the anger that comes out of fear. If I’m very prickly and standoffish, no one will want to be my friend, which is good, because then you can’t reject me. You know? I can’t be hurt if there’s no one close to me to begin with. So, I always kind of had that feeling of being alone. Struggled with anxiety, depression, anger before I started using. I started using because it made me feel a part of, that people invited me to do something. I was like, “Oh my god, they’re inviting me?“ Looking back, I’m like, “Oh my god, [I was] miserable” [113].

Many participants drew a direct line between their lack of social connection, and lack of community connection more broadly, as how they made sense of their substance use. These participants described the role of substances as a tool to make badly needed connections that (almost always) became a source of alienation from more positive connections. For example, one participant explained:

“One of the reasons that I started using drugs and I gravitated towards the friend group that I did was because drugs are what we had in common. As long as I had drugs, I was a part of the group…And today…I’m active in my local community…And that connection is super important to me. I feel connected to that group…whereas when I was actively using drugs, there was no connection. I didn’t have anything to be a part of” [102].

While many participants described having trouble making social and emotional connections with others or being bullied, some participants described feeling disconnected based on demographic characteristics. One participant described class-based differences between him and his peers:

“So, I’m like the burn-out, overachiever kid. So, middle school, obviously all A’s. I went to a private school for a couple of years, which furthered my feelings of alienation, because we were a middle-class family. We made it work. But everyone else there, they had the Hollister and the Aeropostale and the $80.00 shirts. I think that’s where I started to really put up those defense mechanisms because I felt so less there” [113].

Although the sources of social disconnectedness were diverse, the effects were described similarly as perpetuating feelings of isolation and often resulting in needs that participants sought to fill through use of substances.

#### Assets

 We identified two main themes related to assets from participants’ reflection on their earlier lives: skills/talents (theme 4) and relationships (theme 5; see Table 5). In terms of skills/talents, several participants described themselves as gifted across many different areas. For example, a participant described, “I have never done anything halfheartedly.… I’ve probably had 5,000 hobbies and reached a very high level in most of them" [102]. Other participants identified particular areas of interest where they excelled, for example in sports such as basketball, baseball, and cheerleading (with some earning college scholarships for these), and in characteristics such as being socially skilled, highly self-reliant, or enterprising. For example, one participant described being very independent and self-reliant, “I had two jobs. I paid for my own car. I paid for my own phone. And I bought my clothes. My parents– they basically just fed me. They were really proud of me and thought I was doing good things” [128].

Many participants discussed one or more close relationships, and many of these described close family relationships. For example, a participant described “I had a great relationship with my parents. They were very involved. My mother didn’t work until [later], so, we had a lot of time with her…she took us to school and picked us up…My mother was very, very involved” [128]. We note some important nuance across examples of this code; participants seemed motivated to see their family relationships as positive. In some examples, a description of a positive family relationship was quickly followed by explanation of ways in which the relationships seemed strained, for example “My family tries to be supportive, but they do it in such a negative way because they don’t say things to give me good advice. They’re basically judgmental and they’re pushy and they’re not really considerate of my mental health” [121]. For others, descriptions that we coded as positive relationships used fairly neutral language, for example, “Good relationship. The typical teenage wanting your space and such. That sort of phase, I think, was very normal for me and normal for a lot of people” [105]. This may partially reflect that most participants have been through extensive therapeutic programs in their recovery. For example, several participants mentioned repairing relationships with parents and being able to see them in a more positive way, understanding now that their family members may have been doing the best that they could and were perhaps dealing with their own trauma. One participant described a complex relationship with her father, who she described as emotionally abusive but also present for her as a teen:

“…I mean I accept that that’s who he is today. I accept that he’s had a lot of trauma and his way of dealing with it is you put it in a box and you act silly and you act goofy, you just don’t talk about feelings. But he’s a very kind, gentlehearted man. I don’t think he knows how to process feelings” [113].

A nuanced understanding of the complexity of strengths and risk present in the lives of study participants is important because it enables us to see how substance use is not only happening in the context of traditionally recognized “risk” factors such as chaotic households or among young people who feel hopeless or do not feel like they fit in, but also among high-achieving youth and those who describe having some supportive relationships. This finding indicates the need for expanding prevention efforts to include AYA’s not fitting into typical “risk” frameworks.

#### Simultaneous challenges/assets

Interestingly, we also noted several examples where a characteristic of the participants’ personalities or lives served as both risk factors and assets, often simultaneously (theme 6). For example, we identified a theme around being high-achieving and high-performing as sometimes enabling people to use substances because they could and/or they enjoyed the thrill of “doing it all.” For some participants, being “gifted” was discussed as a risk because they did not have to work hard at academics and other skills came naturally to them. One participant discussed being highly driven, which on its face seems more like a protective factor, but also as a risk because they channeled their ambition into understanding, using, and selling substances. Another participant described being highly invested in by others as a risk factor because it created an unfair dynamic with siblings and created pressure for them to perform and succeed. Finally, this came up in the context of being above reproach of parents or other adults; a participant described that they were getting straight A’s, so no one suspected there was a problem. One participant described being a people pleaser as both risky and protective. On the one hand, they wanted to have a good reputation among adults in the community, which deterred them from substance use, but at some point they found that using substances made them popular among their peers. Being religious and having a sense of purpose are sometimes considered protective factors; however, both came up in our interviews as also serving as risk factors. Religion played a complicated role for one participant who felt angry at God when she experienced a death in her family. A different participant described having a sense of purpose/goal early in life that did not involve the need for school, which prompted them to disengage from school: “[School] was just not my thing. I did not want to be there. I knew what I wanted to do with the rest of my life and I didn’t think that a high school education was important for it” [120]. Finally, changes in access to substances came up as both protective and risky. Several participants noted that pills disappeared from the street which prevented further use of them, but resulted in at least one participant turning to heroin use, highlighting how changes in access and substance availability influence substance use.

## Discussion

Our goal for this study was to identify experiences, through retrospective accounts, that YAs in recovery from OUD associate with their past opioid use. Because the “supply side” issues with opioid use – such as limiting the quantity of medications prescribed for pain and curbing access through medication disposal programs – have been the focus of many prevention efforts [2], this study adds perspectives focused on the “demand” side, asking YAs in recovery to reflect on risk and protective experiences along their pathway to opioid use. Our main findings suggest that risk and protective factors were complex; mental health issues and difficulty coping with negative emotions are potential signals of distress and need for support and social disconnection is experienced by many individuals. Below, we discuss these findings and consider implications for understanding and preventing opioid use.

### Beyond risk and protective factors

While a risk and protective factor framework for prevention is valuable in its focus on modifiable factors to prevent substance use at multiple levels of the social ecology, identifying risk factors at the individual level can unintentionally overemphasize individual’s responsibility to “better” deal with adversities and challenges, while ignoring the broader social conditions. Thus, we frame main findings not as individual risk factors but instead as “microsocial factors” [24] or potential signals of social problems and indicate that young people in distress need support. If, as our analysis suggests, opioid use is sometimes a response to cope with experiences of trauma, mental health challenges, negative emotional experiences and social disconnection, it is important to consider how to lower the risk of exposure to such events and conditions. In developmentally-supportive contexts, children should have access to a supportive network of peers, families, and communities. Individuals who experience mental health issues and/or have difficulty coping with negative emotions should be provided with resources, treatment, and support. These social structures are critical to support young people’s healthy development and reduce their exposure to OUD risk factors. Aligned with a social determinants of health framework, opioid use may be viewed at least partly as stemming from insufficient supports in the multiple contexts and systems in which young people are embedded [24, 34]. We suggest that policies and systems need to be re-imagined to support healthy youth development. For example, cross-sector collaborations between health, educational, and community contexts can identify and support young people who are struggling. Indeed, prevention interventions delivered in educational settings, such as those focused on cognitive-behavioral skill building, may promote healthy development of self-regulation skills, thereby reducing risk of using opioids [35]. Brief screening in clinical settings can help identify adolescents who need more intensive support and refer them to appropriate services [36].

In addition, our findings regarding simultaneous risk and protective factors complicate the idea of dichotomous risk and protective factors and point to the need for more nuanced understanding of the ecology of people’s lives and pathways to opioid use. For example, personality traits such as being “high-achieving” and “people-pleasing” might seem to be protective on their face; however, participants discussed how these characteristics sometimes decreased and at times increased the likelihood or severity of their substance use. Applied to prevention, this finding extends prior work by pointing to a need for vigilance even for young people who do not seem “at risk” by traditional measures, and potentially points to a need for tailored prevention programs. Adolescents with different personalities and risk profiles might require different intervention approaches; those who are high-achieving might respond well to interventions that emphasize reflecting on future goals and aligning current behaviors with future goals, whereas those who are sensitive to people-pleasing might respond to interventions that emphasize peer norms and/or rules around substance use.

### Difficulty coping with negative emotions – a signal of distress and need for support

Across both qualitative and quantitative data, the most evident finding pertained to socioemotional development with especially consistent findings that participants perceived that they suffered in terms of *coping with emotions*. From the survey data, a highly noted risk factor was emotional neglect; the mean was notably higher than for the other risk factors measured that pertained to various forms of abuse and physical neglect. One of the lowest endorsed dimensions of protective factors included the item “I talked to my family/caregiver(s) about how I felt.” Together, these findings suggest a need for more support be provided to young people across multiple developmental settings, such as families, schools, and communities. Specifically, more discussion about coping with emotions and broadly about emotional well-being could be helpful within family and school settings.

Aligned with the finding from the survey, one of the main themes we identified from the qualitative data concerned negative emotions and insufficient skills to cope with them. Some participants discussed feeling angry and lonely with little outlet to deal with their feelings. The range of valence of negative emotions in response to discrete experiences and about the self more generally was noteworthy. At the extreme end, some participants described self-contempt and self-hatred (guilt, shame, anger, emotions I didn’t know how to deal with). As is well-documented in the literature on risk for substance use [37] across diverse US racial/ethnic groups [19], including OUD [38], unaddressed mental health issues emerged as a risk factor for substance use among this sample of YAs in recovery from OUD. Our findings align with research findings that opioids are sometimes initiated as a means to cope with complicated psychological and emotional issues, adverse childhood experiences, and life stressors [22, 39].

Findings from the present study support the use of prevention interventions that explicitly attend to issues of coping with difficult emotions, emotional regulation, and social skills and relationships. Indeed, effective individual and family-based prevention interventions include skill building (while prevention programs providing information alone may be less effective) [40]. Some prevention programs that focus on mental health and social emotional skills, such as Botvin Life Skills Training [41], Peaceful Alternatives to Tough Situations [42], and Coping Power [43], could be promising approaches to reducing AYA’s risk of developing an OUD. By addressing the emotional needs of young people, these programs may be promising for reducing their risk of developing an OUD.

Further, these findings have implications for screening-based approaches in both primary care and school-based settings, as early identification of emotional and behavioral issues and/or trauma exposure may allow for connecting youth to resources sooner and prevent worsening of symptoms and illness that may in turn lead to substance use. Existing clinical screening tools focus on catching early substance use (e.g., Screening, Brief Interventions, Referral to Treatment; Simple Screening Instrument for Substance Abuse; Alcohol Use Disorders Identification Test; Opioid Risk Tool, [44, 45]) and some more general tools capture negative emotions or behaviors (e.g. Rapid Assessment for Adolescent Preventive Services, [45]) or identify personality or psychosocial risk factors (Substance Use Risk Profile Scale, Youth Risk Index, [46, 47]). However, findings from the present study suggest that it may be useful for risk assessments to include items about negative self-concept and ability to cope with negative emotions. Further, findings from the present study point to the need for preventive mental health service access and support early and consistently across adolescent and young adult development. In addition to providing access to mental health care, it would be beneficial to prepare young people to benefit from resources such as therapy. On the extreme end, we found a need to prevent, identify, and support young people experiencing trauma and to provide screening and therapy for those with serious mental health concerns. Beyond these acute needs, however, we found a strong need for *all* young people to have access to supportive and caring adults, to develop coping skills to deal with problems, and to learn to name and regulate emotions, especially negative or difficult ones.

### Social disconnection: an individual experience of a social problem

Participants in this study were largely disconnected from positive social relationships with individuals and institutions at the time when they initiated substance use. Although many reported having at least one close relationship, they were often complicated. For example, while some close relations were described as being loving and well-intentioned, they were often also described as being difficult and complicated. When sharing this finding to participants in our feedback sessions, a few noted that despite mentioning a close relationship in their early life, the relationships were not necessarily close *and healthy*. This implies a need for more nuance when examining associations between social connections and substance use [48]. In addition to sometimes strained family relationships, participants noted disconnection from their peers, a lack of support from those outside their families, isolation from school and communities, and a low sense of opportunities for their futures. Aligned with current calls for understanding how social connection relates to health [49], the social disconnectedness we identified is multi-level, best addressed through multi-pronged prevention approaches to building social skills and providing more social opportunities for young people to connect with and contribute to their communities.

### Limitations and implications for future research

Characteristics of the study design and sample should be considered when interpreting results. First, there was no comparison group – this is a sample of YAs in recovery from OUD and most of them identified additional substances that were problematic for them. We do not compare pathways between groups who used different substances and cannot determine based on our design what is uniquely risky for opioids as opposed to other substances in this analysis, although we examined potentially unique motivations and experiences of opioids compared to other substances in a separate analysis (see [50]) Second, this sample is composed of YAs who are highly practiced in reflecting about their substance use and discussing their life through recovery programs and in many cases through mental health counseling and therapy. Our findings, which emphasize the social and emotional risks for OUDs, may partly be picking up on the beneficial effects of this kind of therapy/practice. It is also important to note that we did not explicitly ask about “assets” in our interviews, and thus were not able to directly connect assets to the substance use trajectories of participants. After reflecting on our analysis process for what we may have missed, we determined that we did not adequately capture the positive assets in participants’ lives either through our interview script or through our coding. Driven by our commitment to avoid a stigmatizing view of substance use, and aligned with our beliefs and findings that people who use substances are complex individuals with varied histories and lived experiences, we conducted an additional round of analysis coding for “assets.” Due to the structure of our interview script, it would go beyond the scope of our analysis and findings to over-interpret how assets influence substance use journeys of participants. Indeed, in feedback sessions, study participants noted the stigma associated with addiction, their desire to communicate that opioid addiction can happen to anyone, and the general lack of attention in research to the full humanity of people in recovery.

In this study, our intent was not to generalize findings but rather to generate ideas to inform further prevention efforts targeting opioids, drawing on the experiences of YAs in recovery. Future larger prospective studies should assess the prevalence of social and emotional risk factors such as negative self-concept, lack of social connection, and lack of skills to cope with emotions. Further, studies should examine their interplay with known individual-level risk factors (e.g., use of other substances and traumatic experiences in early life [51]; along with environmental-level risk factors (e.g., poverty, easy access to substances). Although our analysis intentionally focused on the individual level of the social ecology and the psychosocial risk factors for OUD, future work must integrate the individual psychosocial risks within the broader ecological levels that AYAs develop in. The present focus on the individual risk pathways is meant to extend the recent focus on the supply side issues of overprescribing resulting in increased access. Both individual and social factors (such as experiences with trauma, mental health issues, and social disconnection) as well as environmental factors (such as lack of social opportunity and ease of access to substances) play key roles in pathways to opioid use. Importantly, opioid use is often coupled with other substance use and more information is needed about the risks for polysubstance use among AYAs [52–54]; future research can focus on the complexity of motives for single and poly substance use.

## Conclusion

Prevention efforts should strive to “move upstream” by incorporating efforts to reduce preventable trauma, such as sexual abuse and emotional neglect. Environmental efforts targeting harm reduction and “supply side” issues, such as regulating opioid prescribing and limiting access to opioids through medication disposal programs, are important to continue; they should be augmented by a broader “demand side” focus on the complex factors and pathways that lead to opioid use among AYAs. Universal prevention efforts focusing on emotion regulation, mental health, and coping skills may provide high value for *all* young people and targeted help in these areas is needed for those struggling with acute or broader emotional difficulty. Future research should focus on multi-level prevention approaches with recognition that risk and protective factors are complex and may function differently depending on the contexts, opportunities, stages, and situations young people face. Beyond families and schools, policies enabling broader community initiatives are needed to provide support, connection, and opportunities for young people to contribute meaningfully in their communities. Prevention efforts may be improved when the voices of young people, especially those in recovery, are included in the design and implementation of robust prevention approaches.

### Supplementary Information

**Additional file 1.**

## Data Availability

The datasets generated and analyzed during the current study are not publicly available to protect the privacy and confidentiality of participants in this small study (N = 30). Data may be available from the corresponding author on reasonable request.

## Abbreviations

AYA’s: Adolescents and Young Adults
OUD: Opioid Use Disorder
ORT: Opioid Risk Tool
SUD: Substance Use Disorder
US: United States
YAs: Young adults
