# Supplementary material for: Pathways to opioid use and implications for prevention: voices of young adults in recovery
Source: Subst Abuse Treat Prev Policy. 2024 Jan 18;19:8. doi: 10.1186/s13011-023-00584-5 (PMC10795392; doi:10.1186/s13011-023-00584-5)
Supplement: Supplementary file 1 — Additional file 1. [file 13011_2023_584_MOESM1_ESM.docx]

**Interview Script (Set 1) – Adolescents and Young Adults**

**Thank you so much for taking your time to talk with me. My name is [REMOVED], and I am a researcher with [REMOVED. I am trying to understand substance use, especially opioids, among young people. There are no right or wrong answers on these topics, so please just share your honest thoughts and experiences. Your information will be kept confidential or private which means that I will never report your answers connected to your name or organization.**

**Part 1 - Intro, life these days**

To get started, tell me about what your life is like these days?

How do you spend time during the weekdays? During the evenings or at nights? During the weekends?

**Part 2 - Substance use**

**You were introduced to me because you have used opioids at some point. If it’s okay with you, now I have some more specific questions about your experiences with opioids.**

**As a reminder, you don’t have to answer any questions that make you uncomfortable.**

What was your life like at the time when you started using drugs? (Follow up on comments about how life was at school/work, family life, social life, how health was)

What was the first drug or substance you used?

When did you first use opioids?

With any drug, there are things that are enjoyable from the experience, and there are things that aren’t enjoyable about the experience.  I'm curious to hear from you what your experience of opioids was when you first started using.

What drew you to it at first?

What was it like when you first started using?

Did that stay pretty much the same for you, or did the pluses and minuses change for you over time?  How?

Was there anything different that led you to use opioids, compared to other drugs/substances?

Were drugs easy or difficult to access? How did you access drugs? What about opioids specifically? Did you have friends or family or other people you knew using drugs? (If so, which ones?)

Thinking back, what do you remember learning about drugs when you were younger? Do you recall talking about drug use in school or with your family?

Thinking back, were there things or people that might have made you think/feel/act differently about using drugs?

Thinking back, were there things that could have been different that might have prevented opioids from becoming a problem in your life?

Looking back, what you would say to your younger self at the time when you first started using opioids?

**Part 3 - For those who identify as being “in recovery” in screening questions**

**Now I have some questions about what recovery is like for you.**

**Tell me about the process of getting into recovery.**

- How did you know you wanted to stop using drugs?
- Did you get help (formally, like through rehab or another community, or informally like though friends and family)?
  - If you have been in rehab more than one time, what do you think contributed to relapsing?
- What access to help did you have?
- Have you felt supported in recovery?

How is your life different today than it was before you were in recovery?

What has been especially helpful for you in recovery? What has been not so helpful (or things you wish were different?)

Can you think of anything specific that might have kept you from using drugs the first time?

Is there anything you wish people knew about substance use and addiction?

**Part 4 - Project specific**

**I am working on a project thinking about how to include the opinions and perspectives of young people in substance use prevention efforts. The idea has two parts. First, it might beneficial for young people themselves, by providing them with an opportunity to contribute to their communities in a meaningful way. Second, if young people were more involved in prevention strategies and programming efforts, it is possible that the efforts might be more helpful or effective.**

Let’s start with the first idea. What are your thoughts about ways that young people like yourself can contribute to their communities in a meaningful way? How might youth benefit by contributing to their community?

Ok, what about your thoughts on the second idea. How do you think prevention efforts might be more helpful or effective if young people were more involved in prevention strategies and programming efforts?

Have you been involved in any substance use prevention efforts or programs?

(If so, probe for information about the experience: how did you get involved, how long did you participate, did you participate in more than one program, what age were you at the time you participated, what was it like for you)

From your experience, do you have advice for how to maximize the value of young people being involved in prevention efforts?
